# Auxiliary tRNAs: large-scale analysis of tRNA genes reveals patterns of tRNA repertoire dynamics

**DOI:** 10.1093/nar/gku245

**Published:** 2014-04-29

**Authors:** Naama Wald, Hanah Margalit

**Affiliations:** Department of Microbiology and Molecular Genetics, IMRIC, Faculty of Medicine, The Hebrew University of Jerusalem, Jerusalem 91120, Israel

## Abstract

Decoding of all codons can be achieved by a subset of tRNAs. In bacteria, certain tRNA species are mandatory, while others are auxiliary and are variably used. It is currently unknown how this variability has evolved and whether it provides an adaptive advantage. Here we shed light on the subset of auxiliary tRNAs, using genomic data from 319 bacteria. By reconstructing the evolution of tRNAs we show that the auxiliary tRNAs are highly dynamic, being frequently gained and lost along the phylogenetic tree, with a clear dominance of loss events for most auxiliary tRNA species. We reveal distinct co-gain and co-loss patterns for subsets of the auxiliary tRNAs, suggesting that they are subjected to the same selection forces. Controlling for phylogenetic dependencies, we find that the usage of these tRNA species is positively correlated with GC content and may derive directly from nucleotide bias or from preference of Watson–Crick codon–anticodon interactions. Our results highlight the highly dynamic nature of these tRNAs and their complicated balance with codon usage.

## INTRODUCTION

tRNA is a key molecule in all cells due to its central role in protein translation. Each tRNA is an adaptor molecule that can be charged with an amino acid residue and donate it to an elongating peptide chain based on codon–anticodon recognition. Each tRNA species, distinguished by its anticodon sequence, recognizes a specific set of codons, which encode the amino acid it is loaded with. This specificity reflects the genetic code and dictates the translation of a nucleotide sequence into protein. Codon–tRNA interaction exhibits two types of redundancy, whereby the same codon can be decoded by different tRNA species (having different anticodons, but loaded with the same amino acid), and the same tRNA species can decode different codons of the same amino acid. This is achieved by wobble interactions (non-Watson–Crick base pairing) between the third codon position and the first anticodon position. Such wobble interactions can occur due to Guanine-Uracil (G:U) base pairing, as well as many anticodon modifications that change codon specificity (1). Due to this redundancy, not all possible tRNA species are required in order to decode the 61 sense codons, and various organisms employ different subsets of tRNAs (2).

The variability of the tRNA repertoire has been mostly explored in relation to tRNA abundance, reflected by gene copy number. It has been suggested that selection for efficient translation leads to close correspondence between codon usage and the tRNA pool (3). This led to the development of the tRNA adaptation index (tAI) (4), which was used as a measure of translational selection (5–8). In addition, translational selection was found to be associated with the increased number of tRNA genes (9,10). A binary view of an organism tRNA repertoire per se, namely whether a tRNA species exists or not, has been less investigated. In the work mentioned above, Rocha (9) found that translational selection is associated with the decreased number of tRNA species. A survey of the tRNA repertoire in representative organisms from all kingdoms of life led to the formulation of three common strategies employed to achieve reduction in the number of tRNA species, where by each strategy some of the tRNAs were spared (2). Novoa *et al.* (11) showed that archaea employ the smallest number of tRNA species, bacteria prefer the use of U_34_N_35_N_36_ tRNAs (subscript represents position numbering convention where anticodon positions 34, 35 and 36 base pair with codon positions 3, 2 and 1, respectively), in correspondence with the presence of the modifying enzyme tRNA-dependent uridine methyltransferase, and eukaryotes prefer the use of A_34_N_35_N_36_ tRNAs, in correspondence with the presence of the modifying enzyme tRNA-dependent adenosine deaminase. Those studies mainly involved the common patterns of tRNA repertoire at the kingdom level. However, little attention has been addressed to the variations in tRNA repertoire within a kingdom, and it is currently unknown whether these variations are shaped to provide an adaptive advantage.

Here we used a dataset of 319 fully sequenced genus representative bacteria to find patterns of tRNA usage and organism traits associated with them. We found that while certain tRNA species are always used, there is a subset of tRNA species that differs substantially between bacteria. Based on the striking absence of A_34_N_35_N_36_ tRNAs and the extended set of wobble rules, this pattern can be explained by the need to decode all sense codons, thus separating the tRNA species of an organism into mandatory and auxiliary tRNAs. The latter are especially intriguing to investigate, as while an organism can theoretically do without them, different organisms show different repertoires of these tRNAs, and it is not clear what underlies this variability and how it evolved. We use bioinformatic methods employing phylogenetic information to explore the changes in the tRNA repertoire during evolution, with a special emphasis on the auxiliary tRNAs, which were found to be highly dynamic, frequently gained and lost along the phylogenetic tree. Through analysis of sequence similarity, we found evidence pointing at multiple pathways of tRNA species gain through horizontal gene transfer (HGT), anticodon mutations, and a combination of both. Finally, the variability in the use of auxiliary tRNAs was found to be mostly connected to the nucleotide content of the genome, but also to some extent to the strength of translational selection. We discuss possible explanations for the observed associations between the tRNA repertoire and both the nucleotide content and translational selection.

## MATERIALS AND METHODS

### Genome data and organism datasets

The genomic sequence and genome-related data of 1245 bacteria was retrieved from the NCBI FTP site (ftp://ftp.ncbi.nih.gov/genomes/Bacteria, April 2011). To reduce bias from closely related organisms, one bacterium species per genus was randomly selected as representative. We excluded endosymbiotic bacteria with severely reduced genomes (less than 10^6^ bp) and less than 31 tRNA species. This resulted in a dataset of 319 bacteria.

Species tree in Newick format was downloaded from the MicrobesOnline database (12) and was trimmed to the chosen dataset of 319 bacteria. This tree is based on sequence alignment of 78 sets of protein orthologs, encoded by a single copy in most bacteria. While HGT events are common in prokaryotes, this core of conserved proteins represents a central trend of vertical inheritance (13) and can be used to construct a reliable species tree (14).

### tRNA repertoire

Sequences of all non-coding RNAs identified in 319 genus representative bacteria (as annotated by RefSeq) were scanned using the program tRNAscan-SE with bacteria-specific parameters (15). This comprised the repertoire of identified tRNAs and their respective anticodons.

### Association with organism traits

Three genomic traits were considered: genome size, GC content and ENC’_diff_. Genome size and GC content were calculated from the genomic sequence. ENC’_diff_ is an estimate of translational selection at an organism scale calculated as the difference between the average ENC’ of all genes and the genes encoding ribosomal proteins, normalized by the average ENC’ of all genes. ENC′ is a variant of the effective number of codons (ENC) index (16) that accounts for background nucleotide composition (17). ENC’ takes the value of 61 when all codons are used at the frequency expected given the nucleotide composition, and its value decreases as codon usage deviates from the expected. Gene ENC’ was calculated based on the background nucleotide composition of the same gene. The values of the three genomic traits were normalized so that their mean is zero and their standard deviation is one.

We performed regression analysis between the presence/absence profile of each tRNA species and the genomic traits, which takes into account the phylogenetic relationships between the organisms. This was done using the maximum likelihood regression analysis option of the *BayesTraitsV2.0* package (18,19) (http://www.evolution.rdg.ac.uk/BayesTraits.html). While designed for purely continuous data, the algorithm can also be used when the dependent trait is binary. For each presence/absence profile of a tRNA species, the algorithm was used to build regression models with each of the three genomic traits and for each pair of genomic traits, a total of six runs. In addition, the likelihood of the data given only the phylogenetic tree was evaluated by calculating the maximum likelihood when the regression coefficient with each of the genomic traits was set to zero. The parameter λ, which effectively scales the branch lengths of the phylogenetic tree, was estimated in each run in order to allow variation in the strength of the phylogenetic signal. The likelihood calculated when the regression coefficient was set to zero was compared to the likelihood of the regression with genomic trait X to estimate the statistical significance of the contribution of trait X. The likelihood of the regression with genomic trait Y was compared to the likelihood of the regression with both genomic trait X and genomic trait Y to estimate the statistical significance of the contribution of trait X over trait Y. The statistic used is twice the difference between the maximum likelihood values of the compared models. This statistic is distributed as χ^2^ with the number of degrees of freedom that is equal to the difference in the number of parameters between the two models, in this case—one. The association of tRNA presence with a genomic trait was considered statistically significant and independent of the other two genomic traits if adding it to the regression of each of the other traits provided statistically significant results. Since the same data was repeatedly analyzed, false discovery rate procedure was used to control for multiple comparisons based on all tests performed (*p*-value threshold is 8.5 × 10^−3^).

To test whether the statistically insignificant association of some auxiliary tRNAs with GC content stems from the low level of variation in their presence/absence profile, for each of these tRNAs we examined the percentage of genomes that posses it or lack it, and chose the highest of those as a measure of variability. We then compared this measure between the set of auxiliary tRNAs that were statistically significantly associated with GC content and all other auxiliary tRNAs by a Mann–Whitney test.

### Detecting patterns in tRNA repertoire

A binary matrix representing the presence/absence of each tRNA species in 319 bacteria was constructed. The matrix was clustered based on Hamming distance between the presence/absence profiles of each two tRNA species.

The GC content in which an auxiliary tRNA species tends to appear (usage shift) was defined as follows. Bacteria were divided into two groups based on the presence/absence of the tRNA, and each group was ordered by GC content. The GC content where the usage shift occurs was determined as the average between the GC content of the 75 percentile of bacteria in the ‘absent’ group and the GC content of the 25 percentile of bacteria in the ‘present’ group. This procedure was applied only to tRNA species that showed a statistically significant difference in GC content distribution between the ‘absent’ and ‘present’ groups using a Mann–Whitney test.

### Ancestral reconstruction

The GLOOME algorithm (20–22) was used to reconstruct the tRNA repertoire in each internal node of the phylogenetic tree as well as to estimate rates of tRNA gain and loss. The algorithm was applied to 52 tRNA species in all 319 organisms simultaneously (tRNA species never observed in the studied organisms were excluded). The evolutionary model used was ‘variable gain/loss ratio (mixture)’ and gamma rate distribution. Both of these parameters were chosen to allow tRNA species to vary as much as possible in their evolutionary model. We also used high optimization level. The reconstruction assigns a probability for the presence of a tRNA species in each internal node and produces an estimation of gain/loss probability per branch. Nodes were considered as undergoing gain or loss events if the branch leading to them had a gain or loss probability of at least 0.8 (see more details in Supplementary Methods).

### Origin of new tRNA species

Clues as to the origin of a tRNA gene are more likely to exist in recently acquired genes. Most recent gain events (MRGEs) were defined as gain events that have no other gain of the same tRNA species below them in the phylogenetic tree. For a MRGE of tRNA-X, all the genes descending from the gain were compared against a database of tRNA genes from all tRNA species and organisms using BLAST. The BLAST score of a tRNA against itself was used to evaluate the quality of the match. Only hits that scored at least 80% of the self-hit score were further considered. Hits were classified as ‘vertical inheritance’ if they had the same anticodon and descended from the same MRGE as the query, or otherwise as ‘non-vertical inheritance’. For each query, the best-scored hits were considered in the vertical inheritance category and in the non-vertical inheritance category. The results of the ancestral reconstruction, MRGE analysis and the mapping of potential tRNA origins were uploaded to the interactive tree of life (iTOL) (23,24) for visualization in tree context.

### tRNA co-gain and co-loss and operon structure

Operon predictions were retrieved from the MicrobesOnline database (25). Organisms originating from multiple gain events were suspected to acquire the tRNA genes through horizontal operon transfer. For each two tRNA species that had at least one event of co-gain, the number of organisms descending from these events that contain an operon with genes of both tRNA species was recorded. The same was done for organisms not descending from a co-gain event to provide a background of operon co-existence of the two tRNA species.

Nodes where multiple loss events were predicted were suspected to have lost the tRNA genes through operon deletion. We looked for evidence for gene proximity in organisms evolutionarily closest to the co-loss event. Organisms were considered nearest neighbors if they descended from the parent node of the co-loss event but not from the co-loss event itself. For each two tRNA species that had at least one event of co-loss, the number of nearest neighbors that contain an operon with genes of both tRNA species was recorded. The same was done for organisms not considered nearest neighbors to provide a background of operon co-existence of the two tRNA species.

## RESULTS

### Patterns of tRNA usage across species

**Figure 1.**
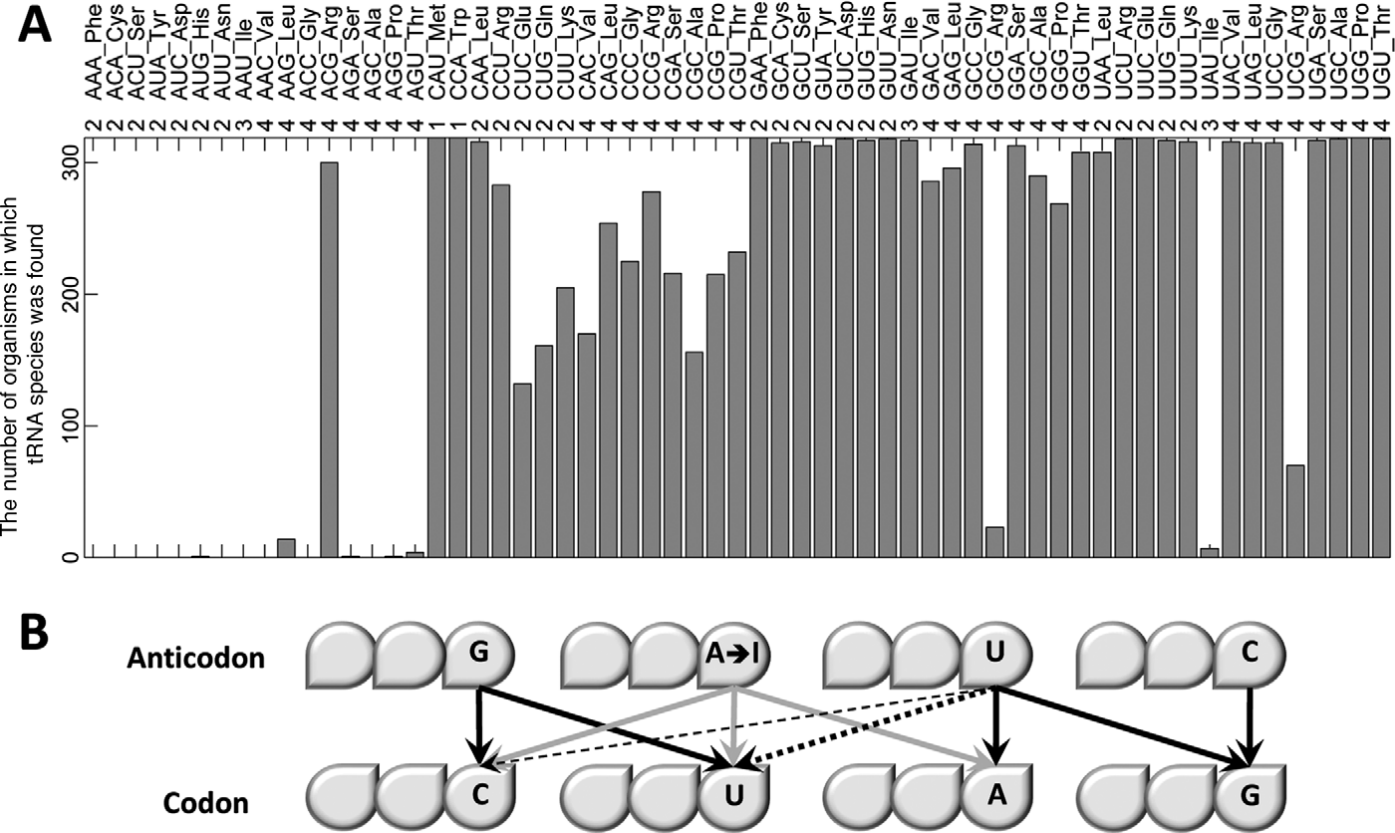
The tRNA repertoire is highly variable. (**A**) The number of organisms (out of 319) that have at least one copy of a tRNA species. The numbers above the bars represent the number of codons in the relevant codon quartet that code for the amino acid decoded by the tRNA. (**B**) A schematic representation of known codon–anticodon interactions. Black solid arrows represent commonly occurring interactions. Gray arrows represent possible interactions when Adenosine is modified to Inosine in the first anticodon position. Black dashed arrows represent modifications-dependent interactions when the first anticodon position contains Uridine. The thickness of the dashed arrows represents interaction efficiency.

We scanned 319 bacterial genomes to identify their tRNA genes (annotated as tRNA species by the anticodon sequence) (Figure 1A). Pseudo tRNAs were excluded. As has been previously reported (2,11) most A_34_N_35_N_36_ tRNA species (except for A_34_C_35_G_36_ tRNA encoding Arginine) were found to be missing from all or the vast majority of organisms. It is known that Adenosine in the anticodon wobble position (position 34) is very efficiently modified to Inosine (I), which recognizes codons ending with C, U and A. This broad specificity has detrimental effects when a codon quartet is split into two pairs of codons (purine- and pyrimidine-ending) encoding different amino acids, as it leads to ambiguities when decoding the A-ending codon. However, when all codons in the quartet encode the same amino acid, this broad specificity is not obviously harmful, as can be seen by the use of such tRNAs in eukaryotes (2). Therefore, while it is not clear yet what underlies the widespread avoidance of A_34_N_35_N_36_ tRNA species in prokaryotes, it seems that there is strong negative selection against them.

The absence of most A_34_N_35_N_36_ tRNAs can explain several observed patterns of tRNA usage. Specifically, U_34_N_35_N_36_ and G_34_N_35_N_36_ tRNA species are present in all, or the vast majority of organisms, since without A_34_N_35_N_36_ tRNAs, both are necessary for decoding the N_1_N_2_A_3_ and N_1_N_2_[CU]_3_ codons, respectively (see the allowed codon–anticodon interactions in Figure 1B). We therefore refer to these tRNA species as mandatory tRNAs. Two other mandatory tRNAs are C_34_A_35_U_36_ and C_34_C_35_A_36_, as they are the sole decoders of the Met and Trp codons. Since the always-present U_34_N_35_N_36_ tRNAs are capable of decoding N_1_N_2_G_3_ codons (Figure 1B), the use of C_34_N_35_N_36_ tRNAs is auxiliary rather than mandatory. Indeed, we found that the rest of the C_34_N_35_N_36_ tRNA species, which decode amino acids with several synonymous codons, show usage variability. Notice that the eight G_34_N_35_N_36_ tRNAs decoding 4-fold degenerate amino acids are probably not as essential as those decoding 2- and 3-fold degenerate amino acids since they can be supplemented or even replaced by the corresponding U_34_N_35_N_36_ tRNA under certain conditions (as discussed below, Figure 1B). Indeed these G_34_N_35_N_36_ tRNAs demonstrate greater usage variability and are therefore considered auxiliary tRNAs as well. There are two major exceptions to the pattern described above. Ile tRNA U_34_A_35_U_36_ is not mandatory but rather rarely seen, since in order to prevent misreading of the Met codon A_1_U_2_G_3_ as Ile, a specially modified tRNA (not considered in this work) exists instead of the conventional U_34_A_35_U_36_ (2). Also, the Arg N_34_C_35_G_36_ tRNA species shows different behavior due to the use of A_34_C_35_G_36_ tRNA by most bacteria. In the presence of A_34_C_35_G_36_ (modified to I_34_C_35_G_36_), G_34_C_35_G_36_ tRNAs becomes hardly used and auxiliary (Figure 1A and B). Since I_34_C_35_G_36_ does not decode the C_1_G_2_G_3_ codon, the presence of either U_34_C_35_G_36_ or C_34_C_35_G_36_ is mandatory, but they can be viewed as auxiliary to one another.

While the apparent selection against A_34_N_35_N_36_ tRNAs combined with codon–anticodon binding possibilities determines the mandatory and auxiliary tRNAs, an intriguing question regards the pattern of usage variability of the auxiliary tRNAs in the various genomes and the principles underlying it. To try and answer this question, we looked for associations between the use of each auxiliary tRNA species and several genomic traits. The genomic traits selected represent three aspects hypothesized to influence the tRNA repertoire: genome size, nucleotide content and translational selection. Genome size was shown to be correlated with tRNA gene number (4) and may therefore correlate with tRNA presence as well. The nucleotide content of the genome has a tremendous effect on codon usage, mainly through the third codon position (26,27). Co-evolution of codon usage and the tRNA pool may therefore be reflected as association between the nucleotide content measured by the GC content of the organism and the tRNA pool. The third genome property is the strength of selection for efficient translation, shown by Rocha to correlate with the total number of tRNA genes and to inversely correlate with the total number of tRNA species (9). We used ENC’_diff_ as a measure of translational selection, calculated similarly to the measure proposed by Rocha in his work (see detailed description in Materials and Methods and in (28)). Briefly, ENC’_diff_ is the normalized difference between the average ENC’ of all genes and the average ENC’ of ribosomal genes, where ENC’ is a measure of gene codon bias. The stronger the selection towards efficient translation in highly expressed genes, the higher the codon bias in the highly expressed ribosomal genes compared to all other genes, resulting in higher ENC’_diff_.

We evaluated the association between tRNA repertoire and these traits by a regression model. The generally hierarchical evolution of species implies that the phylogenetic signal should influence to a certain extent the observed phenotypes (29). In other words, closely related species are expected to have more similar characteristics than remotely related organisms simply due to differences in divergence time, which may lead to biased estimates of association between genomic traits (29). It is therefore essential, when looking for associations between organism traits, to take into consideration the phylogenetic relatedness between genomes. To this end, we performed regression analysis using a phylogenetic generalized least-square approach implemented in the *BayesTraits* package (18,19). As suggested by Ives and Garland (30), the values of the continuous genomic traits were normalized so that the calculated regression coefficients will represent effect sizes. Testing the correlation between each two continuous genomic traits while taking into account the phylogenetic tree (downloaded from the MicrobesOnline database (12)) (Supplementary Methods) revealed a statistically significant correlation between genome size and GC content (*R* = 0.305, *p*-value = 2.2 × 10^−8^) and very weak correlations between ENC’_diff_ and both genome size and GC content (*R* = 0.118, *p*-value = 3.8 × 10^−2^ and *R* = −0.176, *p*-value = 1.6 × 10^−3^, respectively)

**Figure 2.**
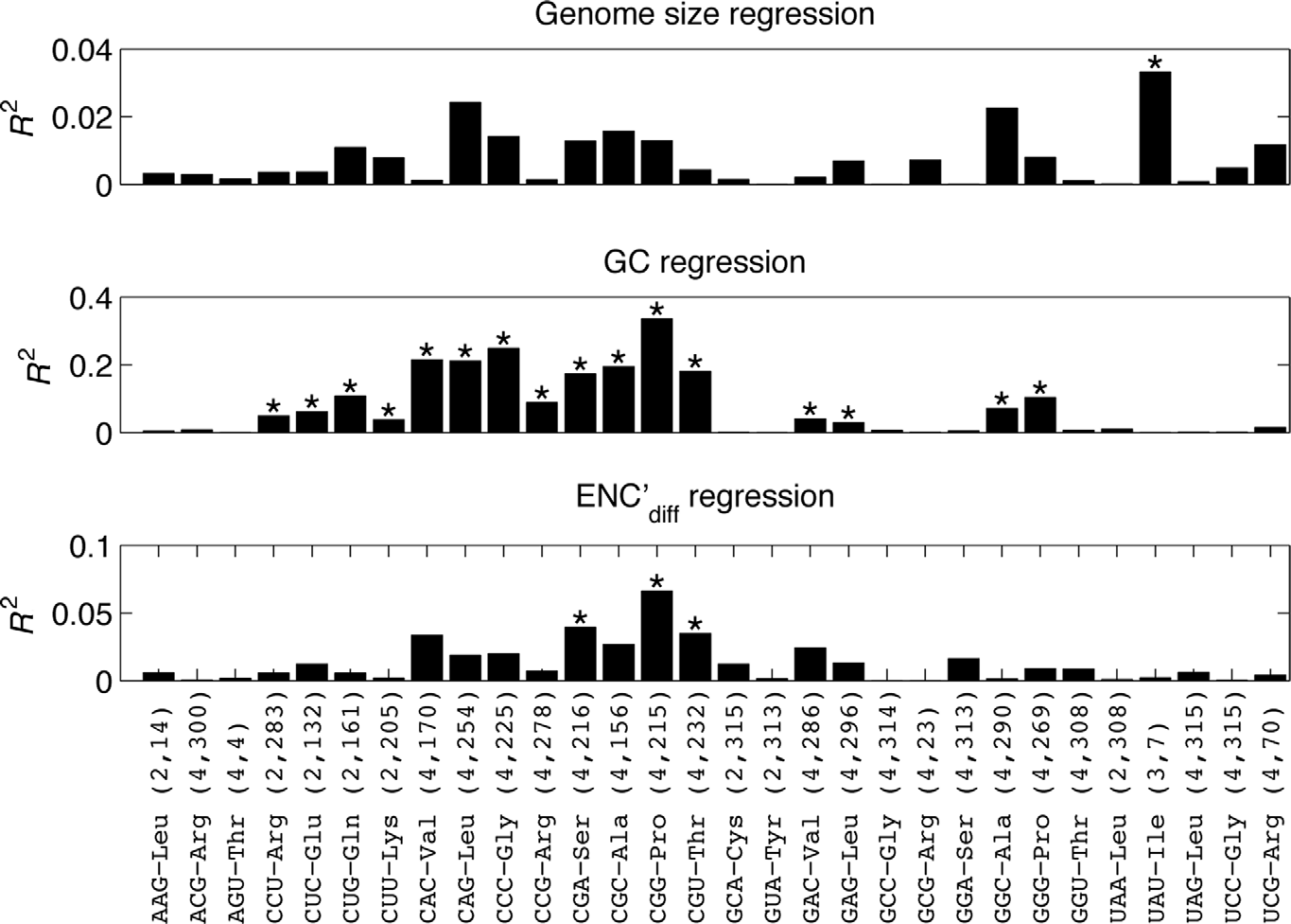
Auxiliary tRNA presence/absence profile is mostly associated with GC content. Regression analysis of tRNA presence/absence profile with genome size (top), GC content (middle) and ENC’_diff_ (bottom). RNA species that exhibited little presence/absence variability (when three or less organisms had a different presence/absence status than the majority of organisms) are not presented. In parentheses are the number of codons in the relevant codon quartet that code for the amino acid decoded by the tRNA and the number of organisms in which the tRNA species was found (out of 319). *R*^2^ is the amount of variation explained by the regression model (note the different *R*^2^ range). Asterisks denote statistically independent genomic traits that when added to regression between the dependent variable (tRNA presence/absence) and each of the additional genomic traits, statistically significantly improved the fit of the data to the calculated regression model.

For each tRNA species, the relationship between its binary presence/absence profile and the normalized values of each of the genomic traits was analyzed, taking into account the phylogenetic dependencies. We allowed the algorithm to simultaneously estimate the parameter λ, which is a measure of the phylogenetic signal. λ values can range between 0 (no phylogenetic signal) and 1 (phylogeny perfectly explains variation). The values calculated for this parameter were often close to one (Supplementary Table S1), indicating a strong phylogenetic signal. It is therefore remarkable that the variation remaining after controlling for the strong phylogenetic signal is often found to be statistically significantly associated with additional genomic traits, as detailed below. Since the continuous genomic traits are not independent, we considered an association of the dependent variable (tRNA repertoire) with trait X to be independent of trait Y if the likelihood of the regression model with both X and Y traits was statistically significantly higher than the likelihood of the regression model with the Y trait alone (Materials and Methods). As can be seen in Figure 2, genome size is not independently associated with the presence/absence status of any tRNA species except for the rarely used U_34_A_35_U_36_-Ile. GC content, on the other hand, is positively (and independently from the genome size and ENC’_diff_ traits) associated with most of the auxiliary tRNAs, explaining between 3 and 34% of the usage variability as measured by *R*^2^. Of note, the auxiliary tRNA species that were not statistically significantly associated with GC content were more uniformly present or absent in bacteria (Mann–Whitney test, *p-value* = 1.3 × 10^−3^, see Materials and Methods). Almost all the auxiliary tRNAs exhibit a negative slope that reflects a negative correlation with ENC’_diff_, although a statistically significant and independent negative association with ENC’_diff_ was observed for only three auxiliary tRNA species (C_34_G_35_A_36_-Ser, C_34_G_35_G_36_-Pro, C_34_G_35_U_36_-Thr). This finding is in agreement with Rocha's discovery that the total number of tRNA species decreases as translational selection increases (9).

**Figure 3.**
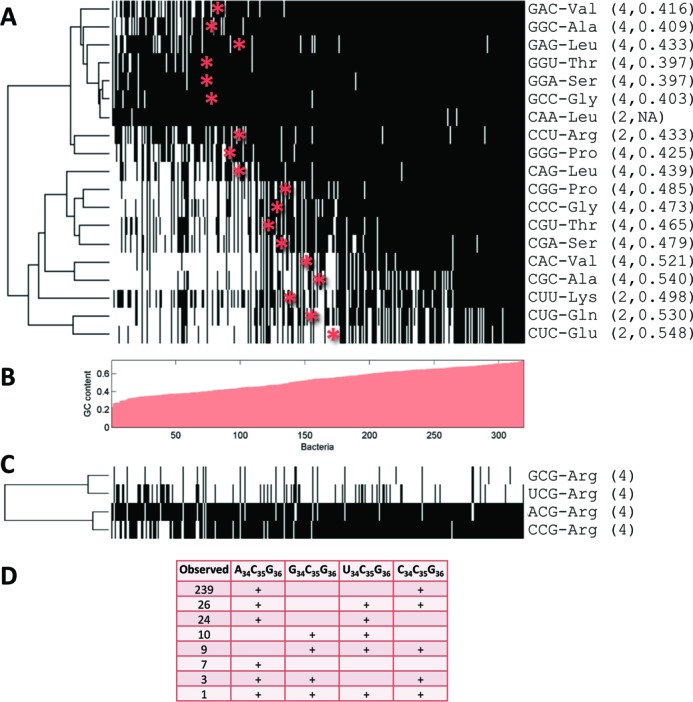
GC content effect on auxiliary tRNA species is variable. (**A**) Clustering of the presence/absence (black/white, respectively) profiles of auxiliary tRNA species in 319 bacteria. tRNA species are clustered based on Hamming distance while bacteria are arranged by their GC content presented in (**B**). The GC content where a tRNA species tends to appear/disappear (usage shift) was defined as the mean between the GC content of the 75 percentile of organisms missing the tRNA and the 25 percentile of organisms that possess the tRNA. Asterisks mark the usage shift location based on B. In parentheses are the number of codons in the relevant codon quartet that code for the amino acid decoded by the tRNA, and the usage shift value, separated by a comma. (**C**) The same as A for the four Arg tRNA species of the type N_34_C_35_G_36_. (**D**) Distribution of Arg tRNA species combinations among the genomes.

We next turned to examine whether the auxiliary tRNAs usage evolved randomly or in a certain determined order. To address this, we presented the data as a matrix of tRNA species over organisms, where each matrix cell contains information about the presence/absence of tRNA X in organism Y. We clustered this matrix on the tRNA dimension using Hamming distance, which measures the number of differences between the profiles of each pair of tRNA species (Figure 3A). Organisms were arranged by their GC content (Figure 3B) as this property was found to be dominant in its relation to the tRNA profile. As can be seen in Figure 3A, there appears to be an order of auxiliary tRNA gain or loss. G_34_N_35_N_36_ and C_34_N_35_N_36_ tRNAs form separate clusters, with the exception of C_34_A_35_A_36_-Leu and C_34_C_35_U_36_-Arg, which are included in the G_34_N_35_N_36_-cluster. In general, the usage shift (Materials and Methods) of G_34_N_35_N_36_ tRNAs occurs in consistently lower GC-content values than that of their C_34_N_35_N_36_ counterparts (Wilcoxon signed rank test *p-value* = 0.0156).

The codon quartet of Arg (tRNA N_34_C_35_G_36_) is an interesting special case as it is the only case where the A_34_N_35_N_36_ tRNA is regularly used (Figure 3C and D)_._ Since A_34_C_35_G_36_ modified to I_34_C_35_G_36_ does not decode the codon C_1_G_2_G_3_ (see Figure 1B), an additional tRNA is required. In most bacteria (239) this is achieved by C_34_C_35_G_36._ However in 24 bacteria the additional tRNA is U_34_C_35_G_36_, and in 26 bacteria both tRNAs are employed. Of note, C_34_C_35_G_36_ tRNA is used when GC content is high and U_34_C_35_G_36_ when it is low. Furthermore, the rare event of supplementing the A_34_C_35_G_36_ tRNA with G_34_C_35_G_36_ tRNA occurs only when GC content is high. In the infrequent absence of A_34_C_35_G_36_ tRNA, both G_34_C_35_G_36_ and U_34_C_35_G_36_ are required to decode the quartet. In nine out of 19 cases, the C_34_C_35_G_36_ tRNA is also found but only when GC content is high. It would appear that even in this more complicated case of tRNA combinatorics, the rule governing the use of auxiliary tRNAs is that G_34_N_35_N_36_ and C_34_N_35_N_36_ are enlisted when GC content is high and the non-essential U_34_N_35_N_36_ is enlisted when GC content is low.

### tRNA dynamics is dominated by gene loss

**Figure 4.**
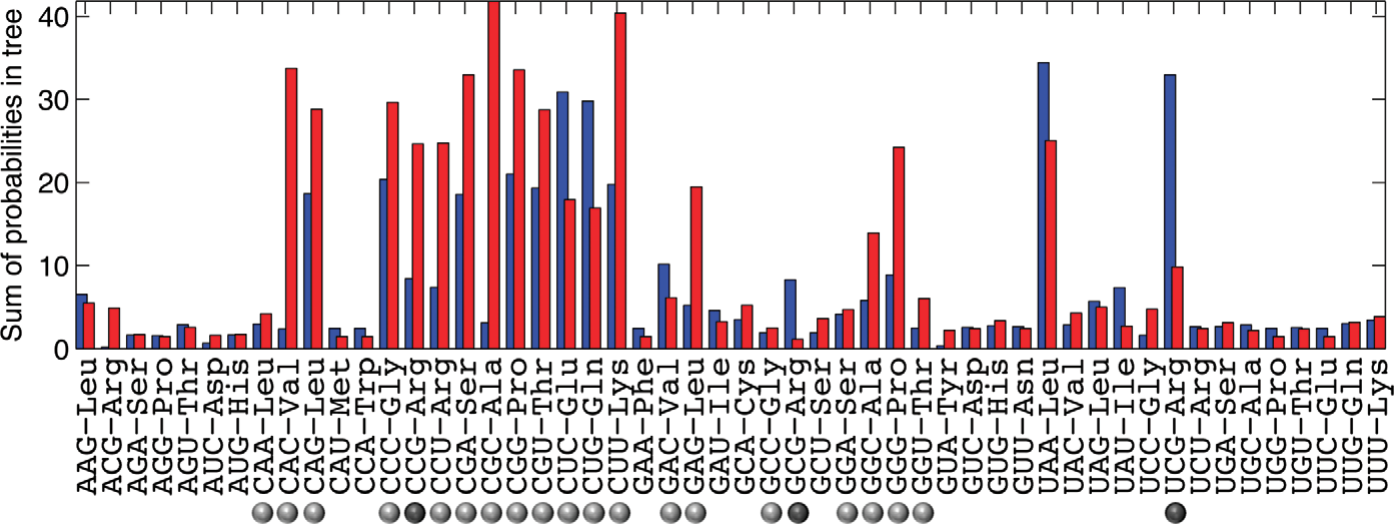
Auxiliary tRNA evolution is highly dynamic and dominated by gene loss. Sum of gain (blue) and loss (red) branch probabilities over the phylogenetic tree calculated by GLOOME. tRNA species are listed alphabetically by their anticodon sequence. Light gray dots mark auxiliary tRNA species. Dark grey dots mark Arg auxiliary tRNA species.

To better understand the dynamic processes that shape tRNA usage, an evolutionary reconstruction is essential. We used the GLOOME software (20–22) to combine tRNA presence/absence profile with the phylogenetic tree and thus reconstructed the probable evolutionary history of each tRNA species. GLOOME implements a stochastic mapping approach to assign gain and loss events onto each branch of a phylogenetic tree based on the topology and branch lengths of the tree. The algorithm was used to infer branch-specific and tRNA-specific gain and loss events using an evolutionary model that permits the gain/loss ratio to vary among tRNAs, thereby allowing for different evolutionary behaviors for the various tRNAs. While the expectation of gain/loss per branch depends on its length, the overall tRNA gain/loss rates are the sum of gain/loss expectations predicted per branch over all the branches of the tree and are therefore comparable (Figure 4). As expected, mandatory tRNAs have very low loss and gain rates. A notable exception is U_34_A_35_A_36_-Leu where a scenario of multiple loss events that occurred late in evolution and a scenario of few early losses followed by multiple gain events both explain the tRNA pattern and are therefore summed together to produce high gain and loss rates. Compared to mandatory tRNAs, auxiliary tRNAs tend to have much higher gain and loss rates. While the rates are highly varied, the dominant trend is of tRNA loss. Only four auxiliary tRNAs are gained at a higher rate than they are lost. These include the rarely used Arg tRNAs G_34_C_35_G_36_ and U_34_C_35_G_36_ that are gained as a replacement when the commonly used A_34_C_35_G_36_ and C_34_C_35_G_36_ are lost, and the highly dynamic C_34_U_35_C_36_-Glu and C_34_U_35_G_36_-Gln. GLOOME calculates a probabilistic reconstruction of ancestral states (see example in Supplementary Figure S1). While there is a distinct signal of vertical inheritance demonstrated by the clusters of related bacteria, all either possessing or missing a specific tRNA, it is also obvious that multiple gain and loss events have occurred repeatedly and independently throughout evolution for most auxiliary tRNAs. The ancestral reconstruction of all auxiliary tRNA species suggests with high probability that they were present at the bacterial ancestor (Supplementary Methods and Supplementary Table S2), as occurs often in extant GC-rich bacteria. This may point to a GC-rich ancestor or to an underlying environmental influence in which the extensive tRNA set promoted survival. Furthermore, this implies that tRNA species with low gain rate like C_34_A_35_C_36_-Val and C_34_G_35_C_36_-Ala once lost are not gained again, whereas for tRNA species with higher gain rate, the loss is not as final.

Identification of gain and loss events makes it also possible to assess the dependencies between the different tRNA species. We used random simulations (Supplementary Materials and Methods) to generate a background distribution of independently gained and lost tRNA species, to which the actual distribution of gain and loss events was compared. This revealed that the observed distribution is statistically significantly biased towards nodes originating from multiple gain or loss events (Supplementary Figure S2), pointing to strong dependencies between tRNA species. Analysis of the individual dependencies between each pair of tRNA species (Supplementary Figure S3) revealed that C_34_U_35_C_36_-Glu and C_34_U_35_G_36_-Gln are co-gained in 10 nodes out of a maximum of 14 (such a result or higher was never reproduced in 10^4^ random simulations, Supplementary Methods). C_34_C_35_C_36_-Gly, C_34_G_35_A_36_-Ser, C_34_G_35_G_36_-Pro and C_34_G_35_U_36_-Thr also tended to be co-gained with each other more than expected at random. Statistically significant co-loss is found in almost all C_34_N_35_N_36_ auxiliary tRNAs and to a lesser extent in G_34_N_35_N_36_ auxiliary tRNAs (mainly in G_34_G_35_C_36_-Ala and G_34_G_35_G_36_-Pro).

A detailed examination of sequential gain and loss events (Supplementary Methods and Supplementary Figure S4) revealed that there are also certain tRNA species that appeared in a specific order whenever they are proximal in the phylogenetic tree. Remarkably, these tRNA species (i.e. C_34_G_35_G_36_-Pro, C_34_G_35_U_36_-Thr, C_34_C_35_C_36_-Gly, C_34_G_35_A_36_-Ser and C_34_U_35_G_36_-Gln) are the same tRNA species that tended to be co-gained. While the results were not statistically significant due to the small number of instances of each type, they form a coherent hierarchy of tRNA gain. Loss event order was less clear and loss hierarchy could not be established. It is possible that the selective pressure on tRNA loss is stronger than the selective pressure on tRNA gain or that tRNA loss is more easily achieved than tRNA gain. This can lead to rapid gene eliminations that appear simultaneously.

### tRNA gain through horizontal gene transfer and anticodon mutations

**Figure 5.**
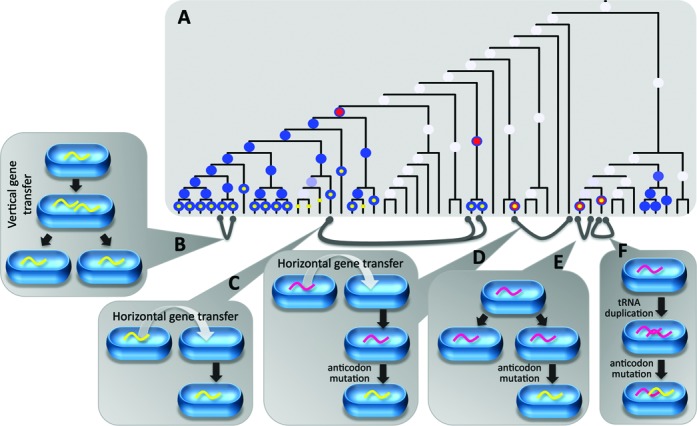
tRNA sequence similarity may reveal the origin of newly acquired tRNA genes. (**A**) A schematic example of tRNA-X ancestral reconstruction. The shade of the blue dots indicates the probability of tRNA-X presence (zero probability is not presented, low probabilities appear white). Red dots mark nodes of MRGEs, where tRNA-X gain is predicted with probability of at least 0.8, and no additional gain event of tRNA-X is found below it in the tree. Yellow dots mark all the currently extant organisms descending from an ancestor where an MRGE occurred. The grey lines connect two organisms demonstrating high tRNA gene sequence similarity according to BLAST. (**B–F**) Mechanisms most likely to explain tRNA gene similarity: (**B**) Vertical gene transfer can explain similarity of two tRNA-X genes (yellow curly lines) descending from the same MRGE. **(C**) Horizontal gene transfer can explain similarity of two tRNA-X genes descending from different MRGEs. **(D**) Horizontal gene transfer of tRNA-Y (magenta curly line) followed by mutations transforming tRNA-Y to tRNA-X can explain similarity of tRNA-X and tRNA-Y genes from distantly related organisms. (**E**) Similar to D but in closely related organisms. Due to vertical gene transfer, closely related organisms have a copy of tRNA-Y. A mutation that transforms tRNA-Y to tRNA-X in one organism creates very similar orthologs of different tRNA species. **(F**) Gene duplication followed by anticodon mutation creates very similar paralogous genes that encode different tRNA species.

tRNA dynamics, while dominated by gene loss, also includes gain events. An interesting question regards the source of new tRNA genes in a genome. Possible mechanisms are HGT and anticodon mutations of already present tRNA genes. Tracking gene origin is difficult due to genetic variation that accumulates with time and erases sequence-embedded evidence. tRNA genes are especially problematic since in such short sequences every mutation eliminates a significant evolutionary signal. Furthermore, since tRNA function largely depends on its tertiary structure, structure-preserving mutations are more easily accumulated. It was shown in bacteria that genes of the same tRNA species are conserved in only 60–85% of their sequence (31). However, since sequence similarity rapidly deteriorates, the finding of highly similar sequences probably hints at a common ancestor that was shared not long ago. In order to find such events, we identified the MRGEs of each tRNA species (see schematic example in Figure 5A), and all the tRNA genes that descended vertically from them, and ran a BLAST search of these genes against a database containing all tRNA genes from the 319 bacteria. Since only strong sequence resemblance is likely to suggest a shared origin, the BLAST score cutoff was set at 80% of BLAST score of the query against itself (maximal score), which roughly corresponds to 3–4 mismatches or 1–2 gaps for a typical tRNA gene. Importantly, this procedure is aimed at identifying with high confidence the origin of new tRNA species. It does not provide a comprehensive inventory of HGT of tRNA genes or anticodon mutations as it misses events where gene copy number is changed (not considered as MRGE) or multiple mutations have accumulated (old events that do not pass the strict threshold). Sequence similarity was attributed to vertical inheritance if the query and hit tRNA genes had the same anticodon and descended from the same MRGE (Figure 5B) and to non-vertical inheritance otherwise. For each query, the highest scored hits in each of these two categories were considered. Since a query could show high similarity both to tRNAs belonging to the same clade (explained by vertical inheritance) and to tRNAs of distant organisms (suggesting HGT), this procedure allowed the detection of the source of a horizontally transferred tRNA to an ancestor of a bacterial clade.

**Figure 6.**
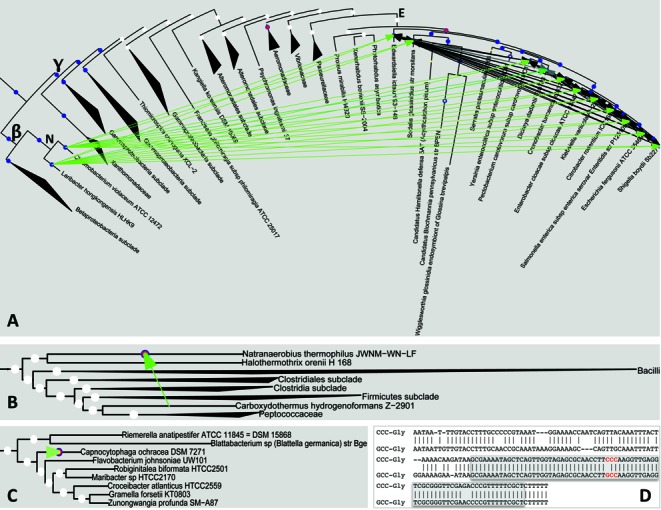
tRNA genes are acquired by multiple pathways. (**A–C**) Similarities between tRNA genes can explain how tRNA genes are acquired. Tree visualization is as described for Figure 5A. Arrows connect organisms that have similar tRNA genes. The arrows are black if the similarity is explained by vertical inheritance and green if by non-vertical inheritance. Clades not involved in gene transfer are collapsed (black triangles). (**A**) C_34_G_35_U_36_-Thr tRNA genes in the *Enterobacteriaceae* family show similarity within the family and are similar to C_34_G_35_U_36_-Thr in the *Neisseriaceae* family, suggesting horizontal gene transfer of C_34_G_35_U_36_-Thr from the *Neisseriaceae* family to the *Enterobacteriaceae* family, followed by vertical gene spread. β, γ, E and N mark the clades of the *Betaproteobacteria* and *Gammaproteobacteria* classes and the *Enterobacteriaceae* and *Neisseriaceae* families, respectively. **(B**) The A_34_C_35_G_36_-Arg tRNA gene in *Carboxydothermus hydrogenoformans* (taxonomy ID: 246194) is the most similar gene to the new U_34_C_35_G_36_-Arg tRNA in *Natranaerobius thermophilus* (taxonomy ID: 457570), suggesting horizontal gene transfer followed by an A to T mutation in the first anticodon position. (**C**) G_34_C_35_C_36_-Gly tRNA gene in *Capnocytophaga ochracea* (taxonomy ID: 521097) is the most similar gene to the new C_34_C_35_C_36_-Gly tRNA of the same organism, suggesting that the G_34_C_35_C_36_-Gly tRNA gene underwent duplication followed by a G to C mutation in the first anticodon position. **(D**) Alignment of the two tRNA genes discussed in C. The duplication/mutation theory is supported by similarities in the sequences flanking the genes. The anticodons of both genes are marked in red.

Out of 403 BLAST hits that passed the threshold, the vast majority (345) was explained by vertical inheritance. We therefore concentrated on the other 58 results. In 53 out of 58 non-vertical inheritance results the query and hit had the same anticodon, but did not descend from the same MRGE event (Figure 5C), which suggests a HGT event. Twenty-two of them are presented in Figure 6A and appear to represent a single HGT event. In this example, an MRGE of C_34_G_35_U_36_-Thr tRNA was found in a clade belonging to the *Enterobacteriaceae* family. This gain event appears to be relatively recent since 11 of the organisms in the clade (out of 13 that did not lose the C_34_G_35_U_36_-Thr gene) also show a strong vertical inheritance signal, which indicates they had little time to diverge. These tRNA genes are very similar to two C_34_G_35_U_36_-Thr genes of the *Neisseriaceae* family (thus creating the 22 non-vertical BLAST hits), suggesting that C_34_G_35_U_36_-Thr tRNA gene was horizontally transferred from an organism in the *Neisseriaceae* family to the *Enterobacteriaceae* ancestor. This explanation is further supported by the fact that organisms in both families are host-associated (most are human pathogens) providing ample opportunities for sharing genetic material.

The second gain mechanism mentioned above is through mutation in the anticodon of an already present tRNA gene that transforms it from one tRNA species to another. There are three possible scenarios involving anticodon mutation: (i) tRNA gene is horizontally transferred and then undergoes anticodon mutation (Figure 5D), resulting in distantly related organisms having very similar tRNA genes that differ in their anticodon. (ii) A mutation occurs in a tRNA gene, transforming it to a different tRNA species (Figure 5E), resulting in closely related organisms having very similar orthologs that differ in their anticodon. (iii) A mutation occurs in a tRNA gene after a duplication event occurred (Figure 5F), resulting in very similar paralogs that differ in their anticodon. We identified two instances of the first scenario and three instances of the third scenario. Figure 6B and C show representative cases of each scenario. In Figure 6B, an MRGE of U_34_C_35_G_36_-Arg tRNA was identified in *Natranaerobius thermophilus* (taxonomy ID: 457570) and showed the highest similarity to the A_34_C_35_G_36_-Arg tRNA gene of *Carboxydothermus hydrogenoformans* (taxonomy ID: 246194), which belongs to an entirely different order and is only distantly related. The evolutionary distance points to a HGT and mutation rather than just a mutation. Interestingly, the presumed gene donor is an anaerobic hyperthermophile isolated from a hot swamp whereas the gene acceptor is an anaerobic thermophile isolated from a solar-heated lake. While not sharing similar tastes in pH and salinity, it is possible that at one time these organisms, their ancestors or close relatives shared the same environment and exchanged genes. The MRGE of C_34_C_35_C_36_-Gly tRNA in *Capnocytophaga ochracea* (taxonomy ID: 521097, Figure 6C) was found to be most similar to the G_34_C_35_C_36_-Gly tRNA gene of the same organism. This suggests that the original G_34_C_35_C_36_-Gly gene was duplicated and then mutated from G to C in the first anticodon position. This explanation is supported by sequence similarity mostly upstream to the gene, that was not found with any other tRNA gene, as would be expected from gene duplication (Figure 6D).

These results demonstrate that tRNA genes can be gained through HGT, anticodon mutations or a combination of both. The fact that we did not find any cases of anticodon mutation without gene duplication (Figure 5E) may suggest that these genes are added to, rather than replace, existing tRNA genes, thereby reinforcing the accessory role of the gained tRNAs. Remarkably, in all five cases where an anticodon mutation was thought to explain tRNA gain, the original and the new tRNA genes always decoded the same amino acid (U_34_C_35_C_36_-Gly/C_34_C_35_C_36_-Gly, G_34_C_35_C_36_-Gly/C_34_C_35_C_36_-Gly, U_34_U_35_G_36_-Gln/C_34_U_35_G_36_-Gln and twice A_34_C_35_G_36_-Arg/T_34_C_35_G_36_-Arg). This is not surprising considering that the original genes are built to be recognized by the correct aminoacyl tRNA synthetase, and few if any additional modifications are required to achieve a functional tRNA gene with altered codon specificity.

### Operon architecture effect is limited to C_34_U_35_C_36_-Glu and C_34_U_35_G_36_-Gln co-gain

Co-gain or co-loss of tRNA species could occur due to the physical proximity of their encoding genes. It is widely acknowledged that tRNA genes tend to cluster in operons, often accompanied by rRNA genes (32–36). We set out to examine if multiple events of tRNA gene gain or loss can be explained by operon gain or loss. The operon data, downloaded from MicrobesOnline, covered at least 60% of the tRNA genes of most organisms (average of 84%, Supplementary Figure S5A). The low coverage observed in 20 organisms is due to genome version differences that prevented tRNA gene mapping to operons, but is not expected to bias the results. On average, 23.8% of tRNA genes were found in an operon with other tRNA genes (Supplementary Figure S5B). This is lower than expected based on several well-annotated organisms (*Bacillus subtilis 168* - 90.7%, *Escherichia coli K12 MG1655* – 69.8%, *Listeria monocytogenes EGD-e* – 88.1%, *Mycobacterium leprae TN* – 35.6%, *Neisseria meningitidis MC58* – 69.5%, *Pseudomonas aeruginosa PAO1* – 58.7%, *Streptomyces coelicolor A3(2)* – 29.2%; data taken from (37,38) and is probably an underestimation due to the high percent of genes that have no operon annotation. In the event of tRNA co-gain driven by a horizontally transferred operon or part of an operon, the operon structure is expected to persist in descending organisms until blurred by gene gains, losses and translocations. We therefore counted for each two tRNA species, the number of organisms descending from a co-gain event in which genes of both tRNA species were found in the same operon (Table 1). The only tRNA pair for which evidence for a co-gain event through operon transfer was found is C_34_U_35_C_36_-Glu/C_34_U_35_G_36_-Gln. Out of 12 organisms descending from C_34_U_35_C_36_-Glu/C_34_U_35_G_36_-Gln co-gain nodes in which both tRNA species were present and had operon information, in seven organisms both tRNA genes were found in the same operon. All the identified operons contain only the two co-gained genes and in all of them the first gene is C_34_U_35_G_36_-Gln followed by C_34_U_35_C_36_-Glu. The operon sequences were aligned and the sequence differences were used to construct an unrooted sequence similarity tree (Supplementary Figure S6). Operons descending from a co-gain event showed higher similarity among themselves as would be expected from vertical inheritance following horizontal operon transfer. Interestingly, in *Acidaminococcus fermentans DSM 20731* (Taxonomy ID: 591001) a perfect duplicate of the C_34_U_35_G_36_-Gln gene precedes the operon. It is possible that gaining the operon did not satisfy the need of the organism, and a duplication of C_34_U_35_G_36_-Gln was also required.

**Table 1.** C_34_U_35_C_36_-Glu/C_34_U_35_G_36_-Gln tRNA co-gain is associated with operon structure

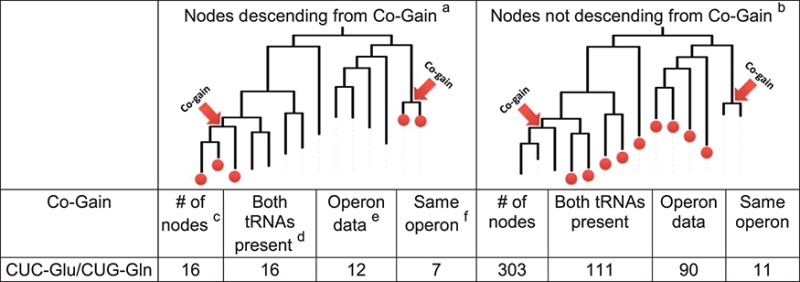

^a^The cartoon is a simplified example aimed at demonstrating the considered nodes. If two tRNAs were gained by horizontal operon transfer, it is more likely that the operon structure will be preserved in organisms descending from the co-gain event.

^b^The cartoon is a simplified example aimed at demonstrating the considered nodes. Nodes not descending from a co-gain event are not suspected to have gone operon transfer and therefore provide a background estimate of finding the two tRNAs in the same operon.

^c^The number of nodes depicted by colorful dots in the cartoon.

^d^The number of nodes in which at least one tRNA gene of each species is present.

^e^The number of nodes in which at least one tRNA gene of each species has operon data.

^f^The number of nodes in which there is at least one operon that contains at least one tRNA gene of each species.

A similar reasoning was applied to co-loss events, where evidence for operon structure was expected to be strongest in organisms closest to the co-loss event. The organisms considered closest are those descending from the parent of the co-loss branch but not from the co-loss branch itself, where unless a gain event has occurred, the tRNA species should be missing (Supplementary Table S3). In seven pairs of tRNA species, each representing only one event of co-loss, at least one of the close organisms had an operon with genes of both tRNA species. However, in all these instances, the co-loss event is accompanied by two to 11 additional tRNA losses that have no evidence for sharing the same operon (details can be found in the text accompanying Supplementary Table S3). It therefore appears that strong selection operating in parallel to eliminate tRNA genes explains the multiple losses better than operon loss.

## DISCUSSION

Analysis of tRNA usage has revealed a subset of tRNA species that we termed auxiliary, since they appear to supplement mandatory tRNA species in some but not all organisms. One of our main aims in this work has been to define and investigate the variability in the use of auxiliary tRNA species in the bacterial world.

### Selection for translational efficiency through tRNA reuse

When examining the changes in tRNA repertoire in regard to ENC’_diff_, a measure of translational selection, we found that the presence of auxiliary tRNA species is weakly negatively associated with ENC’_diff_ (Figure 2 and Supplementary Table S1). While the results were statistically significant independently of both GC content and genome size in only three of the tRNA species, the same trend was observed for most auxiliary tRNAs. These findings are in accord with Rocha's conclusion that translational efficiency is associated with economy in the number of tRNA species accompanied by an increase in their gene copy number (9). We propose that the association of ENC’_diff_ with the reduced number of tRNA species may derive from a more efficient recycling of previously used tRNAs. It was shown for *Saccharomyces cerevisiae* (39), and multiple bacteria (40) and archaea (41) that successive occurrences of the same amino acid favor codons that use the same tRNA. This suggests that reloading a tRNA that has just donated its amino acid to the elongating protein with a new amino acid is more efficient than waiting for a preloaded tRNA to diffuse to the translation site. Thus, if one tRNA species is the sole decoder of a set of codons, it does not matter which codon is used when the same amino acid is encoded next because all codons will be able to reuse that tRNA species. However, when more than one tRNA species is available, the situation is suboptimal. For example, the U_34_N_35_N_36_ tRNA decoding N_1_N_2_A_3_ or N_1_N_2_G_3_ codons can be reused to decode downstream codons of both types, while the auxiliary C_34_N_35_N_36_ tRNA can only be reused to decode downstream N_1_N_2_G_3_ codons, thus reducing translation efficiency. In the case of 4-fold degenerate amino acid decoding, maximal tRNA reuse is achieved when U_34_N_35_N_36_ tRNA is the sole decoder of the entire codon quartet. This type of superwobbling was shown, however, to be less efficient for the U_34_:C_3_ unconventional wobble interaction (42–44) and is therefore expected to be more prevalent when GC content is low, as was indeed observed (2,45,46). As shown in Figure 3, our analysis revealed as well that C_34_N_35_N_36_ and G_34_N_35_N_36_ tRNA species tend to be both absent only when GC content is low.

### The association between the tRNA repertoire and GC content can be explained by selection for Watson–Crick interactions or by nucleotide bias

As GC content decreases, the use of most auxiliary tRNA species decreases. A preference of Watson–Crick over wobble interactions may provide an explanation for these results. While the N_1_N_2_U_3_ codon is always decoded using wobble (either by the commonly used G_34_N_35_N_36_ or by the more rarely used U_34_N_35_N_36_ and I_34_N_35_N_36_ tRNA species) and the N_1_N_2_A_3_ codon is almost always decoded using a Watson–Crick interaction (by the U_34_N_35_N_36_ tRNA species), the N_1_N_2_C_3_ and N_1_N_2_G_3_ codons can either form a Watson–Crick interaction with the G_34_N_35_N_36_ and C_34_N_35_N_36_ tRNA species, respectively, or a wobble interaction otherwise (Figure 1B). The observed coordinated changes in GC content and the tRNA repertoire maximize the number of Watson–Crick interactions by matching the abundant codons with their fully complementary tRNA species. However, except for superwobbling, which was shown to be less efficient than Watson–Crick and wobble interactions, the literature is inconclusive regarding the efficiency differences of wobble and Watson–Crick interactions. Some works claim that standard wobble interactions are less efficient than Watson–Crick interactions (47–50) whereas others find similar efficiency (51,52), and sometimes even increased efficiency (53,54) of wobble compared to Watson–Crick interactions. It was shown that, at least in the case of U_34_N_35_N_36_ tRNA species, efficiency depends on U_34_ modifications (49,54,55), which are unknown for the vast majority of organisms. Furthermore, a clear preference associated with the strength of translational selection was shown for N_1_N_2_U_3_ over N_1_N_2_C_3_ codons in 4-fold degenerate amino acids (28), indicating selection towards wobble rather than Watson–Crick interaction. It is therefore impossible to firmly conclude that Watson–Crick interactions indeed have an advantage over wobble interactions. However, assuming that Watson–Crick interactions are preferred, the much stronger association observed between the tRNA repertoire and GC content compared to ENC’_diff_ implies that Watson–Crick interactions are maximized in organisms independent of the strength of translational selection, and may suggest a basic translation optimization principle observed by all organisms. If this is true, reducing the number of tRNA species in order to improve translation efficiency should not counter the basic mechanism of maximizing Watson–Crick interactions. The ability to balance these requirements greatly depends on the GC content. When GC content is high, a minimal tRNA set that improves tRNA availability reduces Watson–Crick interactions (the abundant N_1_N_2_G_3_ and N_1_N_2_C_3_ codons are decoded using wobble rather than Watson–Crick interactions) and should therefore be discouraged. However, when GC content is low, the minimal tRNA set has little effect on Watson–Crick interactions (the abundant N_1_N_2_A_3_ and N_1_N_2_U_3_ codons continue to be decoded by Watson–Crick and wobble interactions, respectively) and is therefore preferred. Indeed, it is of note that strong translational selection manifested in high ENC’_diff_ tends to occur in organisms with low GC content (data not shown) and may be responsible for the observed weak negative correlation between these two traits (reported above).

An alternative explanation for this phenomenon is that the poorly understood forces shaping nucleotide content (56,57) also influence the nucleotide content of the first anticodon position of tRNA genes. While several studies in both archaea (58) and bacteria (59,60) have shown that the nucleotide content of tRNA genes does not comply with the genomic GC content but is rather restricted by habitat temperature through constraints on folding stability, the wobble position, which does not contribute to structure stability, might be free from these constraints and conform to nucleotide content bias.

Importantly, bacterial GC content does not explain all the variation in auxiliary tRNA usage. It is conceivable that in some cases tRNA genes are gained through mobile elements such as plasmids and prophages and therefore fit the mobile element GC content rather than the GC content of the host bacteria.

### Following tRNA usage through evolution

We explored the changes in tRNA usage throughout evolution by following the tRNA repertoire of a large dataset of bacteria within their phylogenetic context. This powerful approach revealed that auxiliary tRNAs are highly dynamic genes that tend to be lost at a high rate but also have a substantial gain probability. This analysis also provided evidence of complicated dependencies between tRNA species, hinting towards their ordered acquisition. It is, however, not clear what underlies this order. There is no trivial connection between this order and codon–anticodon interaction, as tRNAs with a very similar interaction potential, such as C_34_A_35_G_36_-Leu and C_34_A_35_C_36_-Val or C_34_G_35_G_36_-Pro and C_34_G_35_C_36_-Ala, are not similarly recruited. While it is possible that the order of tRNA recruitment in the various genomes is linked to the abundance of the codons or amino acids the tRNAs decode, which might affect the strength of selection towards their use, such an association was not identified (data not shown).

Due to specific interests of the scientific community and difficulties in maintaining the organism cultures previously required for sequencing, genome sequencing has been highly biased towards certain clades. This resulted in a few dense clades but also many sparse clades, where the tRNA repertoire has changed considerably, but the lack of bifurcations makes it impossible to understand the order of events. Adding more organisms belonging to the genera already represented is not likely to be helpful since organisms in the same genus usually have similar tRNA repertoires. However, it is possible that the rapidly growing coverage of the prokaryotic world, through culture independent and increasingly cheaper genome sequencing, will provide data to better resolve the order of tRNA gain and loss.

Despite the current data limitation, one pair of tRNA species demonstrated a striking dependency in their pattern of appearance. C_34_U_35_C_36_-Glu and C_34_U_35_G_36_-Gln are either present or absent together in approximately 90% of the organisms. They provide the most extreme example of co-gain since out of 14 and 17 gain events found for C_34_U_35_C_36_-Glu and C_34_U_35_G_36_-Gln, respectively, 10 of them co-occurred (Supplementary Figure S6). Also, while less statistically significant, they are co-lost together more than expected by chance. We also show evidence that these two tRNA species tend to be encoded in the same operon. We originally hypothesized that arranging auxiliary tRNAs in operons may be an evolutionary mechanism to facilitate coordinated changes in their genomic occurrence. However, since such arrangement was only observed for C_34_U_35_C_36_-Glu/C_34_U_35_G_36_-Gln, it does not appear that physical proximity is required in order to induce coordinated change of multiple tRNA species. In most bacteria, the glutamine-specific tRNA Gln is first aminoacylated with glutamate, which is converted to glutamine in an amidotransferase reaction requiring adenosine triphosphate (61,62). The frequent clustering of C_34_U_35_C_36_-Glu and C_34_U_35_G_36_-Gln in operons may therefore promote an efficient regulation by their shared amino acid. Incidentally, the ratio of C_34_U_35_C_36_-Glu and C_34_U_35_G_36_-Gln tRNA molecules effectively participating in translation is likely to be lower than of tRNAs of other amino acids since Glu-tRNA is also a precursor of tetrapyrrole pigments (e.g. chlorophylls and hemes) (63–65) and Gln-tRNA amino acid loading is slower due to the additional step of Glu to Gln conversion. It is possible that this reduced yield decreases translational efficiency and is therefore compensated by careful fine-tuning of the tRNA repertoire.

## CONCLUSIONS

The tRNA repertoire can have a tremendous impact on organism fitness. This was mostly shown through the association of tRNA gene copy number and codon usage in relation to the strength of translational selection. However, the evolution and possible effects of the types of tRNA species employed have remained mainly unexplored. We were able to show a weak negative association between the repertoire of auxiliary tRNA species and translational selection and a stronger association with nucleotide content. While the former weak association might indicate an adaptive strategy that promotes translational efficiency, it is unclear whether the latter association is adaptive or neutral. Additionally, by incorporating phylogenetic data, changes in the evolution of the auxiliary tRNA repertoire were traced. This revealed the dynamic nature of auxiliary tRNA species and points to the ease in which such genes can be acquired and lost. We believe that the approach we describe for phylogeny-driven analysis of the auxiliary tRNA repertoire coupled with the continuously growing number of sequenced genomes will provide a better resolution of the dynamics of the tRNA repertoire and determine whether tRNA recruitment is ordered, and if so, which properties underlie this order.

## SUPPLEMENTARY DATA

Supplementary Data are available at NAR Online.

SUPPLEMENTARY DATA
