# Supplementary material for: Auxiliary tRNAs: large-scale analysis of tRNA genes reveals patterns of tRNA repertoire dynamics
Source: Nucleic Acids Res. 2014 Apr 29;42(10):6552–66. doi: 10.1093/nar/gku245 (PMC4041420; doi:10.1093/nar/gku245)
Supplement: SUPPLEMENTARY DATA [file supp_42_10_6552__index.html]

Auxiliary tRNAs: large-scale analysis of tRNA genes reveals patterns of tRNA repertoire dynamics — Auxiliary tRNAs: large-scale analysis of tRNA genes reveals patterns of tRNA repertoire dynamics — SUPPLEMENTARY DATA 

# Auxiliary tRNAs: large-scale analysis of tRNA genes reveals patterns of tRNA repertoire dynamics

## SUPPLEMENTARY DATA

**Files in this Data Supplement:**

- SUPPLEMENTARY DATA
