# Supplementary material for: Auxiliary tRNAs: large-scale analysis of tRNA genes reveals patterns of tRNA repertoire dynamics
Source: Nucleic Acids Res. 2014 Apr 29;42(10):6552–66. doi: 10.1093/nar/gku245 (PMC4041420; doi:10.1093/nar/gku245)
Supplement: SUPPLEMENTARY DATA [file supp_gku245_nar-03195-z-2013-File008.zip › Wald-supplementary-data.pdf]

Supplementary Figures

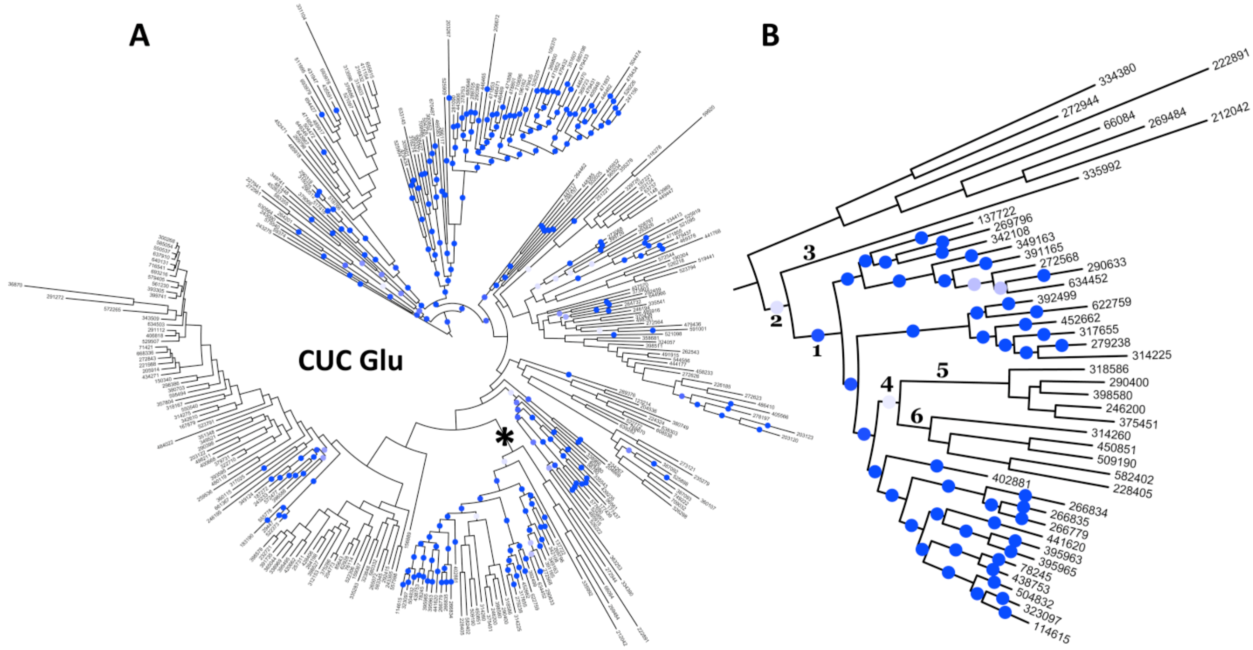

Figure S1 – Example of GLOOME reconstruction results. A) Example of GLOOME ancestral reconstruction of $C_{34}U_{35}C_{36}$-Glu. The blue dots mark the probability of tRNA presence in the node at the end of the branch. The shade of blue marks the probability (the higher the probability the darker the shade. Probability below 0.0625 is not presented). **B**) Magnification of the α-proteobacteria clade (marked with an asterisk in A) shows the probabilistic nature of the reconstructions. Given the nodes below it, it is highly probable that the ancestor numbered **1** had a copy of $C_{34}U_{35}C_{36}$-Glu tRNA. Moreover, considering the adjacent nodes, it is also probable that the gene was gained in that ancestor. However, the algorithm acknowledges that ancestor **2** (the parent of **1**) has a low probability of possessing the tRNA, which can also lead to the current profile, although at a much lower probability (since this requires an additional loss event at ancestor **3**). Similarly, ancestor **4** appears to mark a probable tRNA loss event. However, there is a small probability that the tRNA was present in this ancestor and was lost through at least two separate loss events in its descendents (**5** and **6** or even more downstream).

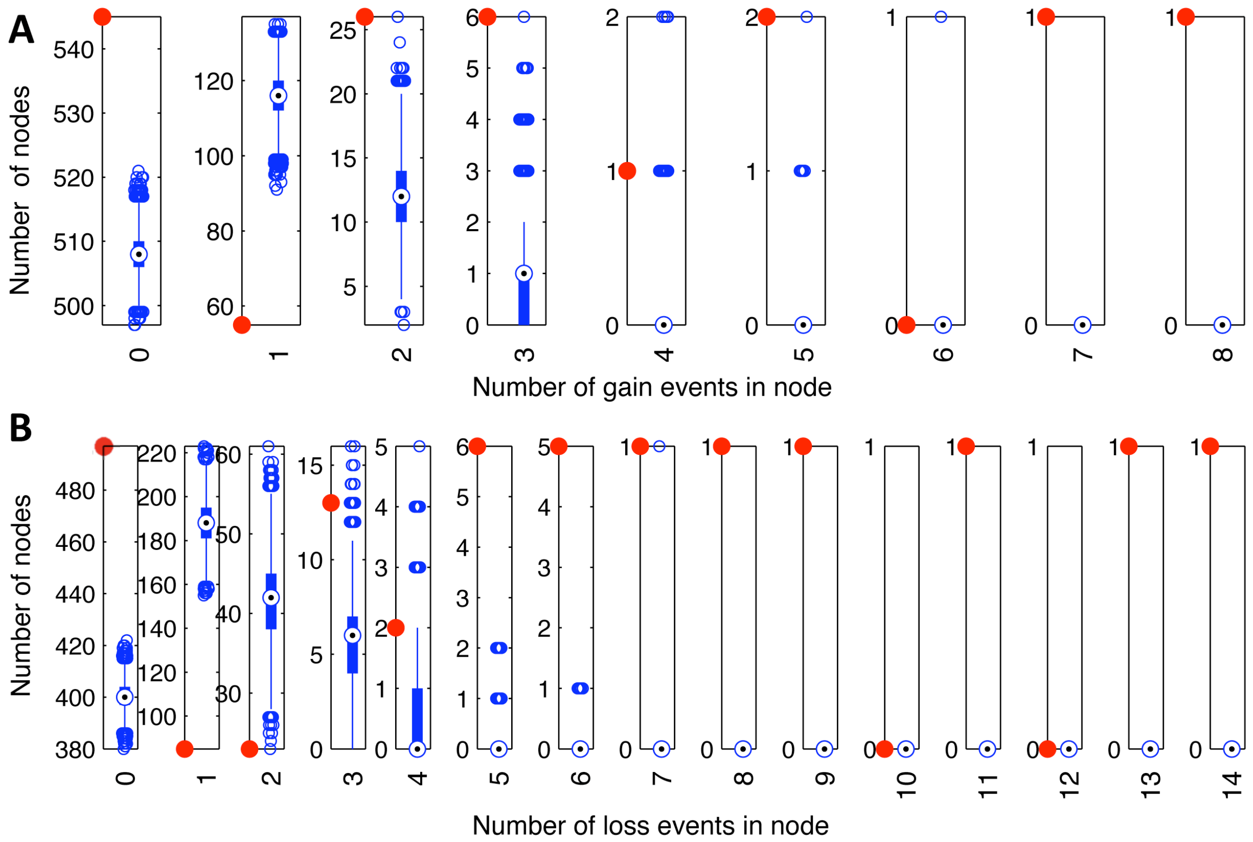

Figure S2 – tRNA gain and loss events tend to cluster in specific nodes. Gain (**A**) and loss (**B**) events of each tRNA species were randomized in the tree (see the Supplementary Methods section below) and the number of nodes in which x gain/loss events occurred was counted. For each x, the distribution of 10^4 randomizations is presented in blue. The results of the non-randomized data are given by the red dot on the left of each plot. Randomizations produce many nodes with few gain/loss events while the real data is biased towards few nodes with many gain/loss events.

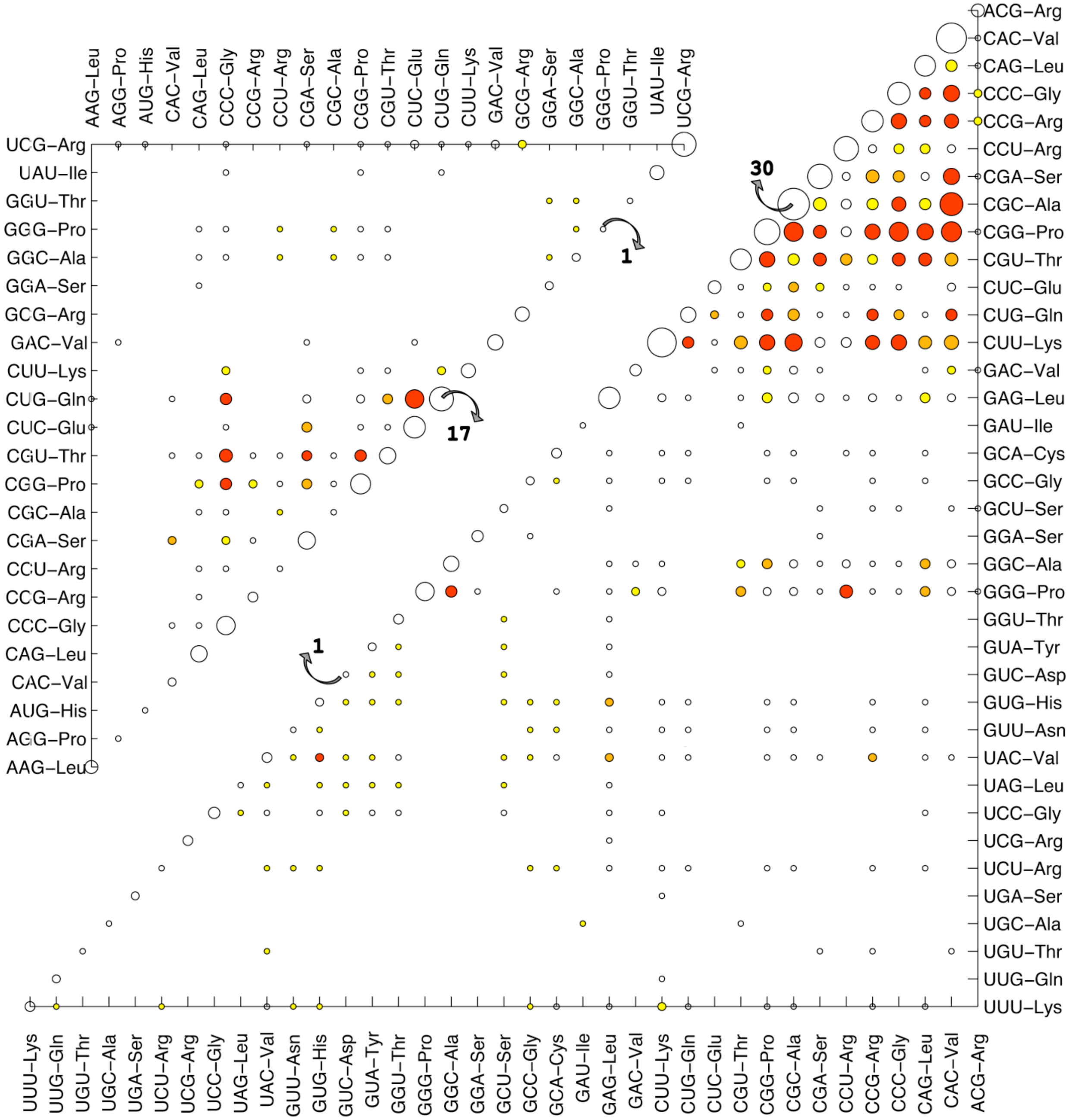

Figure S3 – Certain pairs of the tRNA species tend to be co-gained or co-lost. The intersection of each two tRNA species is marked by a circle proportional in size to the number of nodes in which they were co-gained (upper triangle) or co-lost (lower triangle). The diagonal shows the total number of gains/losses predicted for each tRNA species. The minimal and maximal values are given as reference. Colors indicate the probability of getting such a result or higher if gain/loss events are randomly distributed based on 10^4 random simulations (detailed in the Supplementary Methods section below; yellow<10^{-2}, orange < 10^{-3}, red<10^{-4}). Only tRNA species that were co-gained or co-lost at least once are presented.

A

Event	tRNA-X	tRNA-Y	X->Y	Y->X
gain	CGG_Pro	CGU_Thr	0	6
	CCC_Gly	CGG_Pro	4	0
	CCC_Gly	CGA_Ser	3	0
	CGA_Ser	CGU_Thr	0	3
	CGG_Pro	CUG_Gln	0	3
loss	CAC_Val	CCU_Arg	6	0
	CCU_Arg	CGA_Ser	3	2
	CCU_Arg	CGC_Ala	1	4
	CAC_Val	CGA_Ser	3	1
	CAC_Val	CGC_Ala	1	3
	CGA_Ser	CGC_Ala	0	4
	CGC_Ala	CGU_Thr	4	0
	CAC_Val	CGU_Thr	3	0
	CAC_Val	GAG_Leu	3	0
	CAG_Leu	CGA_Ser	3	0
	CAG_Leu	CGC_Ala	0	3
	CCC_Gly	CGC_Ala	0	3
	CCG_Arg	CCU_Arg	1	2
	CCG_Arg	CGC_Ala	0	3
	CCU_Arg	CGG_Pro	1	2
	CCU_Arg	GAG_Leu	3	0
	CGC_Ala	CGG_Pro	3	0

B

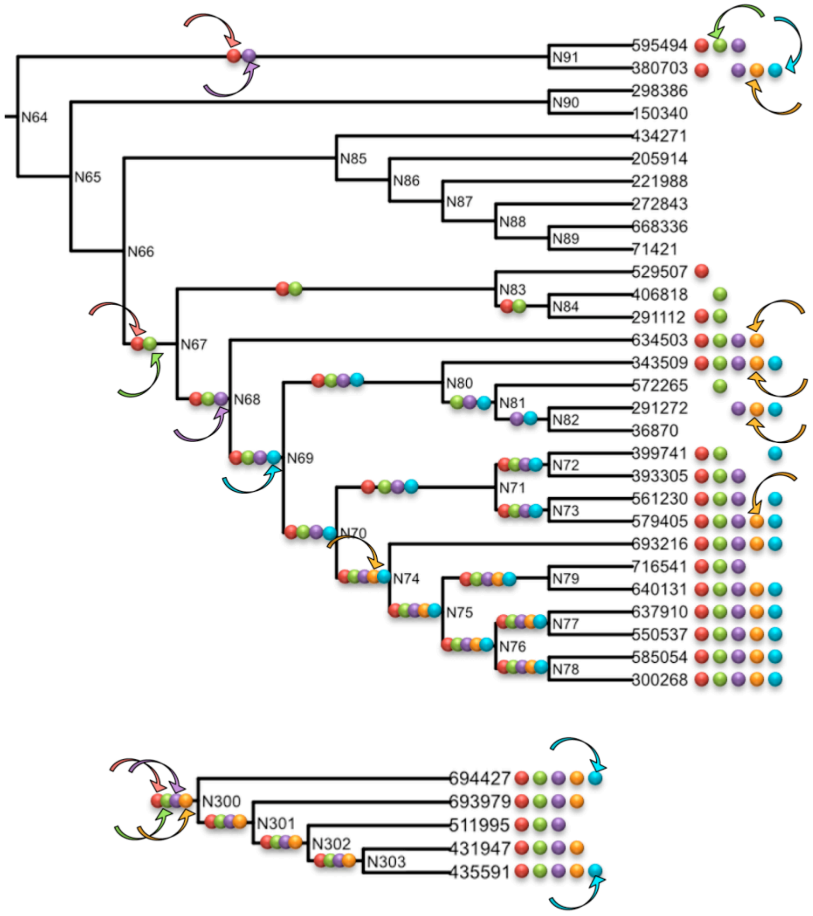

C

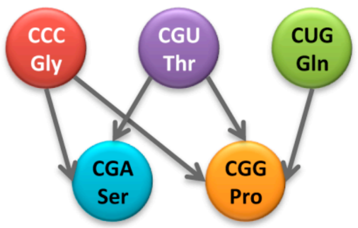

Figure S4 – Evidence of tRNA species gain/loss order. A) Sequential tRNA gain and loss. X->Y: number of tRNA-X gain/loss events followed by tRNA-Y gain/loss. Y->X: number of tRNA-Y gain/loss events followed by tRNA-X gain/loss. Results are shown only if there are at least three sequential events (sum of X->Y and Y->X). **B**) Two examples of sequential gain events. Each colored dot represents the predicted presence of one tRNA species ($C_{34}C_{35}C_{36}$-Gly: red, $C_{34}G_{35}U_{36}$-Thr: purple, $C_{34}U_{35}G_{36}$-Gln: green, $C_{34}G_{35}A_{36}$-Ser: cyan, $C_{34}G_{35}G_{36}$-Pro: orange). Arrows mark predicted gain events. For easier visualization, branch lengths are ignored and organisms are referred to by their taxonomy ID. Not all sequential gain events seen in **B** are counted in **A** due to restrictions on the maximal distance between events (up to 5 nodes apart and 0.82 total branch length). **C**) Schematic representation of tRNA-gain order. The top layer represents tRNA species that tend to be gained before the tRNA species connected to them by arrows in the lower layer.

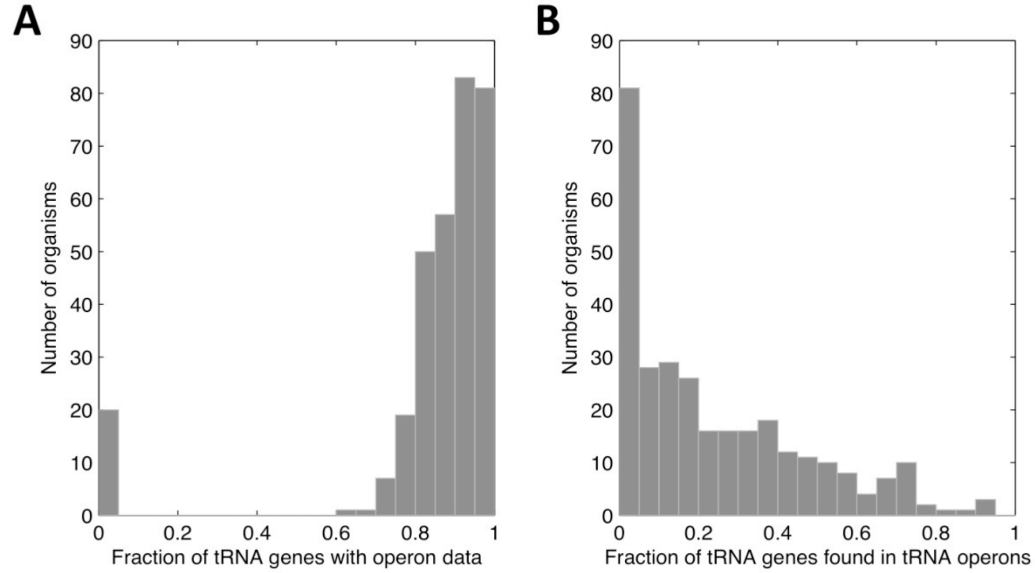

Figure S5 – tRNAs are often encoded within operons that contain other tRNA genes. A) Histogram of the fraction of tRNA genes with operon data. **B**) Histogram of the fraction of tRNA genes predicted to share an operon with other tRNA genes. Based on 299 bacteria for which operon data could be mapped.

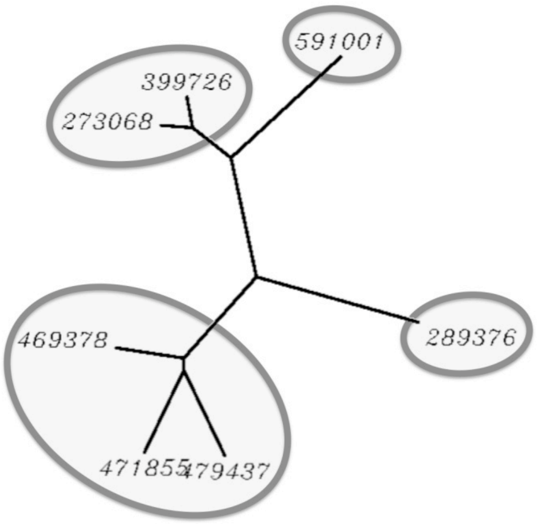

Figure S6 – tRNA genes suspected to be gained by operon transfer have similar sequences. Operon sequences (starting 20 nucleotides before the $C_{34}U_{35}G_{36}$-Gln gene and ending 20 nucleotides after the $C_{34}U_{35}C_{36}$-Glu gene) were aligned using CLUSTALW and the resulting distance matrix was used to calculate an unrooted tree based on neighbor-joining where branches are proportional to sequence distances. Organisms are identified by their taxonomic numbers. The grey ovals surround organisms whose operon sequences are thought to descend from the same co-gain event.

Supplementary table

Table S3– tRNA co-loss is rarely associated with operon structure

	Nodes descending near Co-Loss [a] 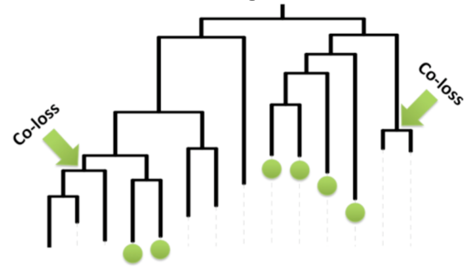				Nodes not descending near Co-Loss [b] 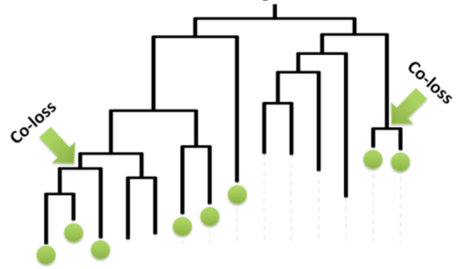			
Co-Loss	# of nodes [1]	Both tRNAs present [2]	Operon data [3]	Same operon [4]	# of nodes	Both tRNAs present	Operon data	Same operon
UCC-Gly / GGU-Thr	1	1	1	1	318	304	239	69
UAG-Leu / GUG-His	1	1	1	1	318	314	247	16
GGC-Ala / CAG-Leu	39	36	26	1	280	208	126	2
CGG-Pro / CGC-Ala	56	46	27	1	263	110	83	0
CGA-Ser / ACG-Arg	318	207	135	3	1	0	0	0
ACG-Arg / CCG-Arg	318	269	177	1	1	0	0	0
ACG-Arg / GCU-Ser	318	298	231	42	1	0	0	0

[a] The cartoon is a simplified example aimed at demonstrating the considered nearest neighbor nodes. If two tRNAs were lost by operon deletion, it is more likely that the operon structure will be preserved in organisms closest to the Co-Loss event.

[b] The cartoon is a simplified example aimed at demonstrating the considered nodes. Nodes directly descending or farther away from the Co-Loss event are less likely to preserve operon structure and therefore provide a background estimate of finding the two tRNAs in the same operon.

[c] The number of nodes depicted by colorful dots in the cartoon.

[d] The number of nodes in which at least one tRNA gene of each species is present.

[e] The number of nodes in which at least one tRNA gene of each species has operon data.

[f] The number of nodes in which there is at least one operon that contains at least one tRNA gene of each species.

The first two pairs in Table S3, $U_{34}C_{35}C_{36}$-Gly/$G_{34}G_{35}U_{36}$-Thr and $U_{34}A_{35}G_{36}$-Leu/$G_{34}U_{35}G_{36}$-His, were co-lost in the same bacteria, *Alteromonas macleodii str. 'Deep ecotype'*. In fact, this organism is predicted to lose a total of nine tRNA species. Since there is only weak support for the simultaneous removal of two pairs of tRNA species, it seems more probable that selection against these genes was strong enough to remove them independently. Similarly, the tRNA pair $G_{34}G_{35}C_{36}$-Ala/$C_{34}A_{35}G_{36}$-Leu was co-lost in the bacteria *Candidatus Pelagibacter ubique HTCC1062* along with 11 other tRNA species, $C_{34}G_{35}G_{36}$-Pro/$C_{34}G_{35}C_{36}$-Ala was co-lost in the

Hydrogenothermaceae family along with two other tRNA species and $C_{34}G_{35}A_{36}$-Ser/$A_{34}C_{35}G_{36}$-Arg, $A_{34}C_{35}G_{36}$-Arg/$C_{34}C_{35}G_{36}$-Arg and $A_{34}C_{35}G_{36}$-Arg/$G_{34}C_{35}U_{36}$-Ser (which constitute four unique tRNA species) were co-lost in *Treponema denticola ATCC 35405* along with two other tRNA species. The latter is a special case since the organism in question diverges directly after the root of the tree, which makes all other nodes in the tree fall under the category of descending near the co-loss event. In all these cases, strong selection operating in parallel to eliminate tRNA genes seems to explain the multiple losses better than operon loss.

Supplementary Methods

Ancestral reconstruction

In order to choose an appropriate threshold of gain/loss probability for our analyses, the probability distribution was examined. As can be seen in the figure below, there is a bimodal distribution and the 0.8 threshold marks the boundary of the more confident set of assignments.

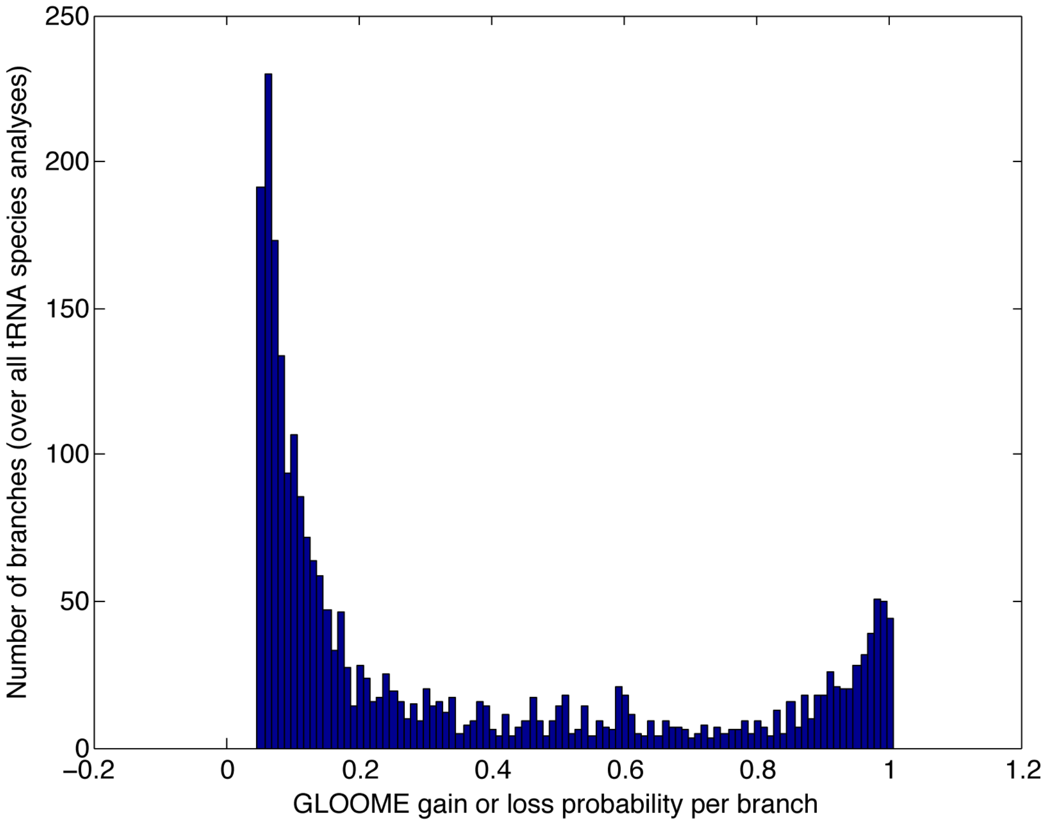

When attempting to use a lower threshold of 0.6, more results were added. For example, the number of instances of co-gained tRNAs increased from 119 to 145. However, when manually inspecting the additional results, it became apparent that many of the low confidence gains and losses are indeed spurious and better explained by other evolutionary pathways. We therefore report results using the more stringent threshold.

Genomic traits correlation

In order to account for the phylogenetic history of the organism set, correlation between each pair of continuous genomic traits across the 319 bacteria was evaluated using the maximum likelihood Continuous (Random Walk) analysis option of the BayesTraitsV2.0 package. The parameter λ, which scales the branch lengths of the phylogenetic tree, was estimated in each run in order to allow variation in the strength of the phylogenetic signal. For each pair of traits, the algorithm was used to estimate the correlation coefficient. The statistical significance of the

correlation was evaluated by comparing the likelihood of the data given the phylogenetic tree and the correlation between the two traits, and the likelihood of the data given the phylogenetic tree *per se* (when the correlation coefficient is set to zero). The statistic used is twice the difference between these two likelihoods, which is distributed as χ^2 with one degree of freedom.

Root estimation

GLOOME and BayesTraits were used to evaluate the probability of tRNA existence in the bacterial ancestor (Supplementary Table S2). GLOOME predictions for the root node were taken from the AncestralReconstructPosterior.txt file created by GLOOME, which contains for each node in the tree the calculated probability for the presence of each tRNA species. The maximum likelihood Multistates analysis option of the BayesTraitsV2.0 package was used to evaluate the root probability for each tRNA species that has at least minimal presence/absence variability (more than 3 organisms differ in their presence/absence from the majority of organisms). The algorithm was run once with default parameters to evaluate the root absence p(0) and presence p(1) probability. In order to calculate the statistical significance of the finding, maximum likelihood was calculated when the root was set to 0 (absent) and to 1 (present). The statistic used is twice the absolute difference between these two likelihoods, which is distributed as χ^2 with one degree of freedom.

Statistical significance of co-occurring gain and loss events

tRNA co-gain frequencies were compared to random distribution of tRNA co-gain generated using simulations. For m tRNA species, m sets of nodes where picked in each run of the simulation so that each set corresponds in size to a set of determined gains of one of the tRNA species. Nodes were chosen randomly with the following constraints: 1) the root node was never chosen since a gain event can never be predicted without an out-group, 2) adjacent nodes were not chosen since between two gain events there must be at least one intervening node with a loss event. The number of nodes found in x sets was counted. The actual number

of nodes where x gains were found was compared to the distribution of the number of nodes that appeared in x sets when the simulation was repeated 10^4 times. The same procedure was applied to tRNA loss.

Simple example:

3 tRNA species are considered (m = 3):

Gain was observed in the following number of nodes: tRNA1 = 10, tRNA2 = 30, tRNA3 = 5.

In each run of the simulation we choose three sets of random nodes according to the constraints described above, where set1 contains 10 nodes, set2 contains 30 nodes and set3 contains 5 nodes.

We than calculate:

Number of nodes that were never chosen (x = 0)

Number of nodes chosen in exactly one of the sets (x = 1)

Number of nodes chosen in exactly two of the sets (x = 2)

Number of nodes chosen in all three sets (x = 3)

After repeating the simulation many times, we get a distribution of values for each x, to which we can compare the actual results. If, for example, there are 5 nodes were all three tRNAs were observed to be gained together, while in only one out of 10^4 simulations such a result was observed, the relevant p-value is 10^{-4}.

To assess the statistical significance of co-occurrence of gain/loss events of a specific pair of tRNA species we used simulations to compute the random probability of such an event. For two tRNA species, one determined to be gained X times, the other Y times and both gained together Z times, we picked in each run of the simulation a set of X nodes and a set of Y nodes. The nodes were selected with the same constraints described above. We then counted how many

nodes in the two sets overlapped (Zr). p-value was calculated as defined by the following equation:

$$pVal = \frac{1}{n}\sum_{i=1}^{n} H(Zr_i)$$

$$H(Zr_i) = \begin{cases} 1; & Zr_i \geq Z \\ 0; & else \end{cases}$$

Where i is the i-th random run and n is the total number of random runs ($n = 10^4$). While the procedure was described for co-gain events, it is identical for co-loss events. The simulations were run for each combination of X and Y observed in the data.

Simple example:

tRNA1 was gained 20 times (X)

tRNA2 was gained 15 times (Y)

tRNA1 was gained with tRNA2 10 times (Z).

In each run of the simulation two sets of 20 and 15 nodes are randomly chosen

Run1: 2 nodes appear in both sets (Zr)

Run2: 1 node appears in both sets (Zr)

Run3: 3 nodes appear in both sets (Zr)

Run4: 0 nodes appear in both sets (Zr)

Run5: 0 nodes appear in both sets (Zr)

…

Run10000: 1 node appears in both sets (Zr)

If out of 10^4 simulations, in two the result was 10 or higher, the calculated p-value is $2/10^4$.

Evaluating order of tRNA gain and loss events

Order of gain and loss events was evaluated by counting the occurrences of consecutive gain or loss events. For each two tRNA species, tRNA-X and tRNA-Y, a gain of tRNA-X followed by a gain of tRNA-Y was counted if the following conditions were met: 1) no additional gain or loss of tRNA-X between the two events, 2) no more than 5 nodes or total branch length of over 0.82 (average branch length x 5) between the two events. The corresponding procedure was applied to tRNA loss events as well.
